# Changes in proportionate cardiovascular mortality in patients with chronic infectious and inflammatory conditions in the United States, 1999–2018

**DOI:** 10.1038/s41598-021-03407-4

**Published:** 2021-12-14

**Authors:** Jacob W. Groenendyk, Adovich S. Rivera, Arjun Sinha, Donald M. Lloyd-Jones, Matthew J. Feinstein

**Affiliations:** 1 Department of Medicine, Northwestern University Feinberg School of Medicine, 680 N. Lake Shore Drive, Suite 1400, Chicago, IL USA; 2 Department of Preventive Medicine, Northwestern University Feinberg School of Medicine, Chicago, IL USA; 3 Institute for Public Health and Management, Northwestern University Feinberg School of Medicine, Chicago, IL USA; 4 Division of Cardiology, Northwestern University Feinberg School of Medicine, Chicago, IL USA

**Keywords:** Cardiovascular diseases, HIV infections, Risk factors, Rheumatic diseases

## Abstract

Treatment options for several chronic infectious and inflammatory conditions have expanded in recent years. This may have implications for evolving competing risks for chronic inflammation-associated comorbidities, including cardiovascular diseases (CVDs). Yet sparse data exist on patterns over time in cardiovascular mortality for chronic infectious and inflammatory conditions. We used data from the Centers for Disease Control and Prevention 1999–2018 Multiple Causes of Death database to investigate patterns in CVD mortality from January 1, 1999 to December 31, 2018 in several infectious and inflammatory conditions. Specifically, we determined age-adjusted proportionate CVD mortality separately for patients with the following conditions (as well as the general population): hepatitis C virus (HCV), human immunodeficiency virus (HIV), inflammatory bowel diseases (IBD), psoriasis (PSO), rheumatoid arthritis (RA), and systemic lupus erythematosus (SLE). Proportionate CVD mortality differed significantly in 1999 and 2018 for each condition compared with the general population (p < 0.0001). Proportionate CVD mortality decreased steadily in the general population (40.9 to 30.6%) but increased for patients with HCV (7.0 to 10.2%) and HIV (1.9 to 6.7%). For IBD, PSO, RA, and SLE, proportionate CVD mortality initially decreased followed by plateauing or increasing rates. Underlying disease-specific pathophysiologies, changes in natural history, and competing risks of chronic end-organ diseases contributing to these differences merit further study.

## Introduction

Diagnostic and therapeutic advances in the past several decades have altered the clinical course and prognosis of many chronic infectious and inflammatory conditions. Novel anti-infectious treatments such as antiretroviral therapy (ART) and direct-acting antivirals have improved mortality in individuals living with human immunodeficiency virus and hepatitis C virus, respectively^1,2^. In chronic inflammatory conditions, development and wider use of immunomodulatory medications has likewise, in some cases, changed symptom burden and expected clinical course. Conversely, recent decades have seen an increasing body of evidence for the contribution of inflammation to cardiovascular disease (CVD) in both the general population and sub-populations with chronic infectious and inflammatory conditions^3–7^. We hypothesized that these changes in treatment of chronic infectious and inflammatory conditions may have implications on evolving risks for cardiovascular disease (CVD) and all-cause mortality. Sparse data exist regarding patterns over time in CVD mortality for chronic infectious and inflammatory diseases (CID), limiting current understanding of changing burdens of CVD (vs. competing non-CVD causes of death) in these conditions. Therefore, we investigated patterns of CVD mortality over time in six relatively common infectious and inflammatory conditions (CID) which, combined, affect approximately 14 million Americans: hepatitis C virus (HCV), human immunodeficiency virus (HIV), inflammatory bowel diseases (IBD, including ulcerative colitis and Crohn’s disease), psoriasis (PSO), rheumatoid arthritis (RA), and systemic lupus erythematosus (SLE)^8–13^. Several of these conditions are associated with higher CVD risk compared with control populations without chronic infectious or inflammatory diseases^4,14–17^. Yet, relatively little is known regarding the overall burden of CVD mortality associated with these conditions, especially given their evolving natural histories (e.g., decreased HCV-related hepatocellular carcinoma/death or improved disease control with biologic therapies for inflammatory diseases) and related changes in competing causes of death.

## Results

### Patterns in proportionate CVD mortality for the general population compared with CIDs

**Figure 1:**
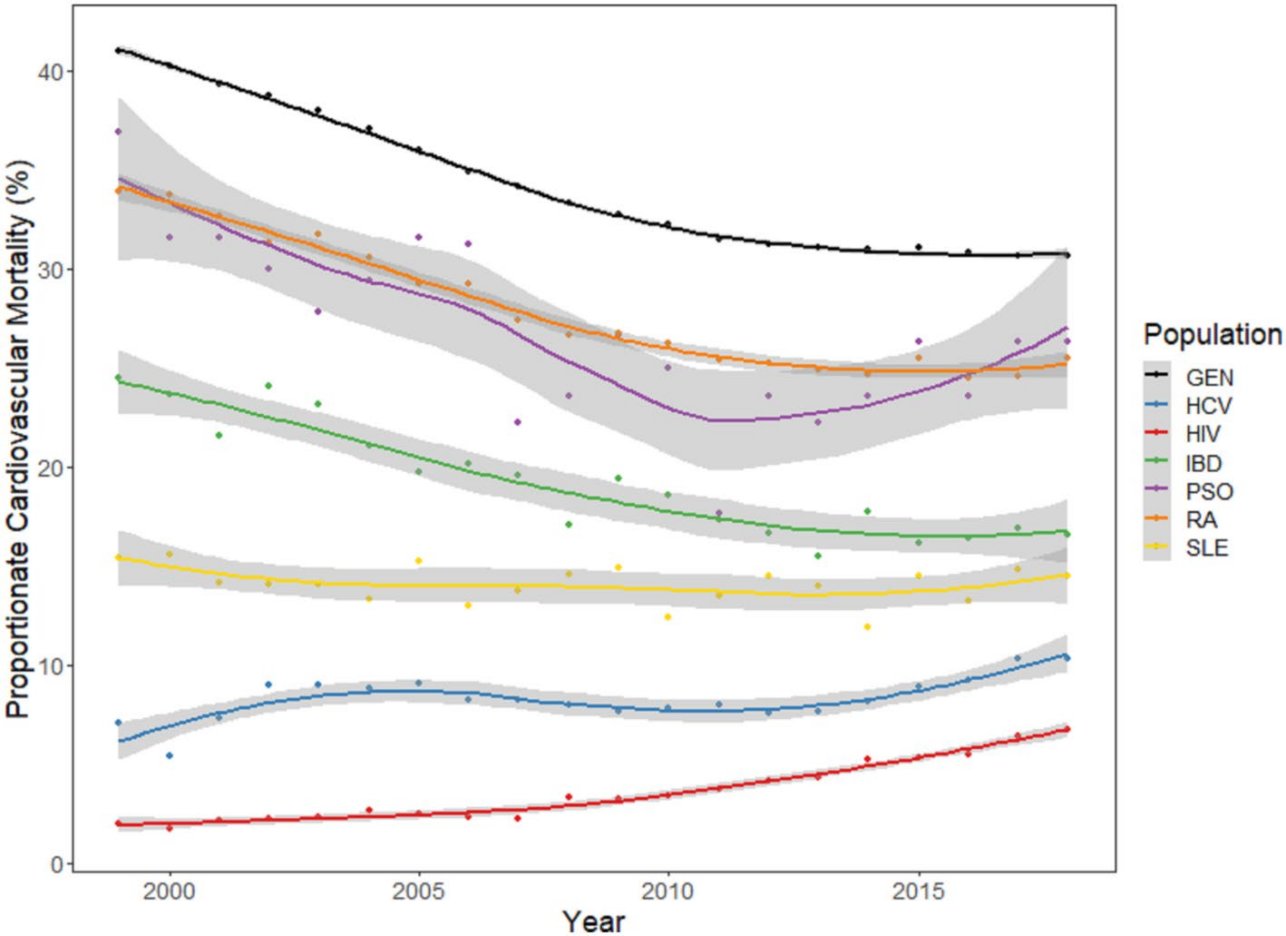
Proportionate cardiovascular mortality over time by disease, 1999–2018. *GEN* General population, *HCV* Chronic Hepatitis C Virus, ICD-10 code B18.2, *HIV* Human immunodeficiency virus, ICD-10 codes B20–24. *IBD* Inflammatory bowel disease, ICD-10 codes K50–51, *PSO* Psoriasis, ICD-10 code L40, *RA* Rheumatoid Arthritis, ICD-10 codes M5–M6, *SLE* Systemic lupus erythematous, ICD-10 code M32.

Proportionate cardiovascular mortality (PCVM) for the general population was significantly higher than each of the CID groups from 1999 (41.0%) through 2018 (30.6%) (Table 1. Changes over time were heterogeneous by specific CID; PCVM for each CID in both 1999 and 2018 differed significantly (p < 0.0001) from that of the general population (Table 1). Figure 1 depicts patterns of PCVM from 1999 to 2018 for the general population and CIDs studied. Whereas PCVM in RA and psoriasis declined largely in parallel to the patterns observed in the general population, PCVM increased substantially from 1999 to 2018 for HCV (7.0 to 10.3%) and HIV (1.9 to 6.7%). Meanwhile, PCVM for SLE remained largely stable from 1999 to 2018 (15.4 to 14.4%).

**Table 1:** Ordinary least squares modeling of proportionate cardiovascular mortality in 1999 vs. 2018 by condition.

CID group	1999			2018		
	PCVM (%)	Absolute difference from general (%, 95% CI)	p-value (GEN)	PCVM (%)	Absolute difference from general (%, 95% CI)	p-value (GEN)
GEN	41.0	–	–	30.6	–	–
HCV	7.0	− 34.1 (− 36.1 to − 32.2)	< 0.0001	10.3	− 21 (− 23 to − 19.1)	< 0.0001
HIV	1.9	− 39.6 (− 41.5 to − 37.6)	< 0.0001	6.7	− 23.8 (− 25.7 to − 21.8)	< 0.0001
IBD	24.4	− 16.8 (− 18.8 to − 14.9)	< 0.0001	16.5	− 14.2 (− 16.1 to − 12.2)	< 0.0001
PSO	36.8	− 6 (− 7.9 to − 4.0)	< 0.0001	26.3	− 4.6 (− 6.6 to − 2.7)	< 0.0001
RA	33.9	− 6.9 (− 8.9 to − 5.0)	< 0.0001	25.4	− 5.7 (− 7.7 to − 3.8)	< 0.0001
SLE	15.4	− 26.4 (− 28.3 to − 24.5)	< 0.0001	14.4	− 16.2 (− 18.2 to − 14.3)	< 0.0001

*GEN* General population, *HCV* Chronic Hepatitis C Virus, ICD-10 code B18.2, *HIV* Human immunodeficiency virus, ICD-10 codes B20–24, *IBD* Inflammatory bowel disease, ICD-10 codes K50–51, *PSO* Psoriasis, ICD-10 code L40, *RHA* Rheumatoid Arthritis, ICD-10 codes M5–M6, *SLE* Systemic lupus erythematous, ICD-10 code M32. All PCVM (and resulting operations) reported as percent. p-values represent test of difference between the general population and each individual CID group within the same year (1999 or 2018).

These visually apparent changes in patterns of PCVM were confirmed by Joinpoint regression (Table 2). In the general population, we observed several inflection points, most notably in 2011, at which point the decline in PCVM plateaued to become nearly flat (β = − 0.1, 95% CI − 0.14 to − 0.07) from 2011 to 2018, compared to β = − 0.64 (95% CI − 0.69 to − 0.59) during 2007 to 2011. At least one join point was present for each of the conditions evaluated, with the year of first join point ranging from 2003 (HCV) to 2014 (SLE); no condition exhibited uniform slope throughout the time period studied. At present, PCVM is increasing for each of the conditions studied.

**Table 2:** Joinpoint regression results.

GROUP	Joinpoints		Slopes per segment				
	Number	Years	Average annual percent change (%)	Slope in segment 1	Slope in segment 2	Slope in segment 3	Slope in segment 4
GEN	3	2003, 2007, 2011	− 0.55	− 0.75 (− 0.8 to − 0.69)	− 1.1 (− 1.2 to − 0.95)	− 0.64 (− 0.69 to − 0.59)	− 0.1 (− 0.14 to − 0.07)
HCV	2	2003, 2013	0.24	0.76 (0.24 to 1.29)	− 0.16 (− 0.29 to − 0.03)	0.58 (0.3 to 0.85)	NA
HIV	2	2007, 2013	0.25	0.08 (0 to 0.16)	0.3 (0.18 to 0.43)	0.45 (0.33 to 0.58)	NA
IBD	1	2013	− 0.39	− 0.58 (− 0.72 to − 0.44)	0.09 (− 0.41 to 0.58)	NA	NA
PSO	1	2011	− 0.39	− 0.58 (− 0.72 to − 0.44)	0.09 (− 0.41 to 0.58)	NA	NA
RA	2	2008, 2013	− 0.42	− 1.06 (− 1.46 to − 0.67)	0.67 (− 0.33 to 1.68)	NA	NA
SLE	1	2014	− 0.49	− 0.8 (− 0.91 to − 0.7)	− 0.47 (− 0.89 to − 0.04)	0.04 (− 0.19 to 0.27)	NA

*GEN* General population, *HCV* Chronic Hepatitis C Virus, ICD-10 code B18.2, *HIV* Human immunodeficiency virus, ICD-10 codes B20–24, *IBD* Inflammatory bowel disease, ICD-10 codes K50–51, *PSO* Psoriasis, ICD-10 code L40, *RHA* Rheumatoid Arthritis, ICD-10 codes M5–M6, *SLE* Systemic lupus erythematous, ICD-10 code M32, *NA* not applicable.

### Differences in PCVM by reported sex and race

Sex-stratified patterns of PCVM over time were generally similar in direction and magnitude for males and females in the general population and within each CID, with patterns for each sex largely mirroring those of the overall population for that specific CID (or the general population: Supplementary Fig. 1). However, there were differences by reported race in PCVM patterns within certain CIDs (Supplementary Fig. 2). Differences by race were most marked in HIV; we observed a significantly steeper rise from 2010 and after for Black individuals with HIV compared with White individuals with HIV. While there was no significant difference in 1999 [Black = 2.0% vs White = 1.9%, difference = − 0.7 (95% CI − 1.6 to 0.1)], this widened by 2018 [Black = 7.6% vs White = 6.0%, difference = 1.0 (95% CI 0.1 to 1.8)]. Likewise, we observed significantly higher PCVM for Black individuals with HCV than White individuals with HCV (difference in 2005: 3.6 (95% CI 2.7 to 4.5) vs difference in 2018: 4.7 (95% CI 3.8 to 5.6)) (Supplementary Table 1). Comparisons were limited to those identifying as White and Black since the other race categories had too few data points for adequately powered comparisons; additionally, psoriasis was not compared due to insufficient data in the Black group.

### Patterns of CVD subtype-specific mortality

**Figure 2:**
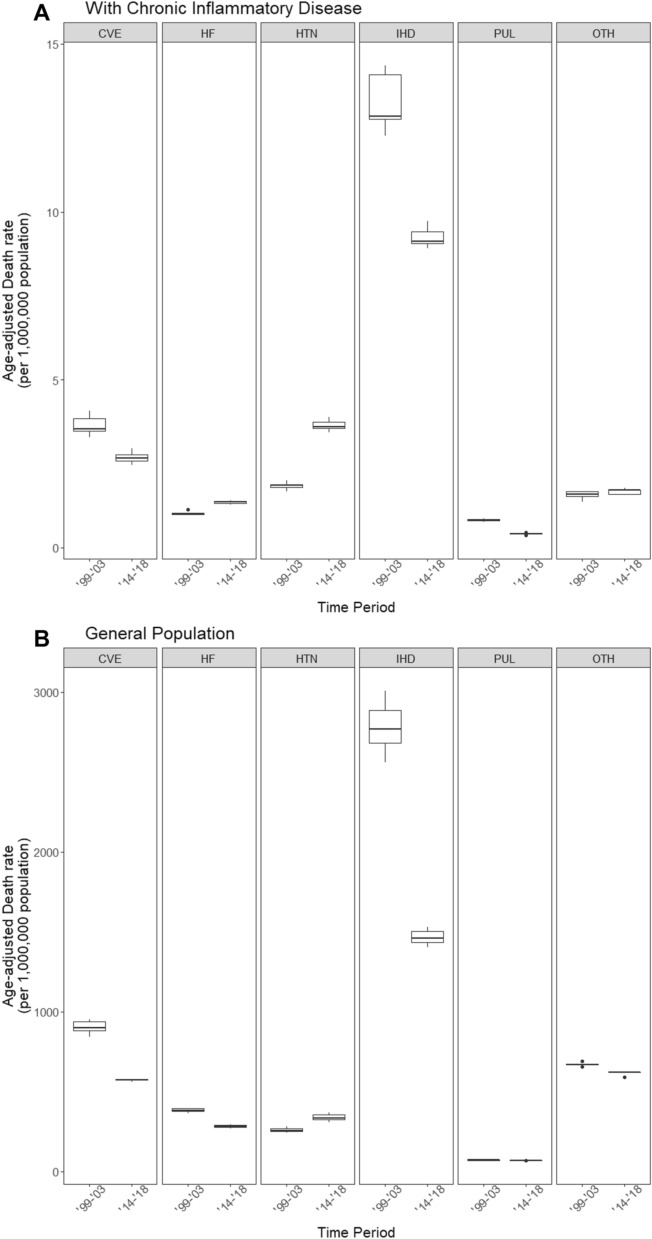
Age-adjusted cardiovascular death rate by cause for patients with chronic infectious or inflammatory disease in 1999–2003 vs 2014–2018. *CVE* Cerebrovascular events, *HF* Heart failure, *HTN* Hypertensive diseases, *IHD* Ischemic heart disease, *OTH* Other causes of cardiovascular disease, *PUL* Pulmonary heart disease.

As expected given general declines in overall PCVM, age-adjusted rates of most subtypes of cardiovascular disease declined when comparing median rates in 1999–2003 with that of 2014–2018. Notably, median rate of ischemic heart disease decreased substantially from 12.8 to 9.1 per 1,000,000 persons. Heart failure-related deaths decreased by a smaller relative magnitude (from 1.0 to 1.4), whereas those associated with hypertensive diseases nearly doubled over the period studied (from 1.9 to 3.6). We did not observe this doubling in deaths due to hypertensive diseases in the general population (from 257 to 333 per 1,000,000) (Fig. 2).

## Discussion

In a nationwide database of death certificate data, we observed significant differences in proportionate cardiovascular mortality (PCVM) patterns from 1999 to 2018 for several common chronic infectious and inflammatory diseases. Whereas patterns of PCVM declined for RA, IBD, and PSO largely in parallel to the general population from 1999 to 2018, PCVM increased significantly for HIV and HCV over the same time period and remained largely stable for SLE. There were also pronounced differences in PCVM by race for certain CIDs, with PCVM increases over time being considerably higher for Black persons with HIV or HCV compared with White individuals.

Several CIDs investigated in this study saw changes in PCVM that mirrored those occurring in the general population. The reported incidence of cardiac death with inflammatory bowel disease or rheumatoid arthritis listed as a MCD decreased by a similar proportion to the decrease in mortality from CVD seen in the general population during our study period. The average annual change in PCVM for IBD and RA was − 0.39% and − 0.49%, respectively, in comparison to − 0.55% for the general population. The PCVM for psoriasis changed by a similar percentage over the entire study period (− 0.42%/year), though interestingly the PCVM initially decreased rapidly (− 1.1%/year) but increased after 2011 (0.67%/year). The lack of difference between the curves of IBD, RA, and that of the general population may reflect limited changes in sources of competing mortality for patients with IBD and RA over the time period studying. It is unclear why PCVM for patients with psoriasis has been increasing since 2011; these findings merit further investigation. We observe little change in PCVM among those with SLE during the study period. It is possible that this is confounded by a changes in the diagnostic criteria for SLE during the study period. The Systemic Lupus International Collaborating clinics announced new guidelines for classification of SLE in 2012, which were more sensitive and less specific than the prior American College of Rheumatology (1997) guidelines. In turn, the European League Against Rheumatism and American College of Rheumatology announced new classification guidelines in 2019^18^. Given the delay associated with widespread uptake of most guideline changes and the fact that most patients with SLE are diagnosed decades prior to death, we believe it is unlikely that these changes in diagnostic criteria substantially altered our findings.

In contrast to patients with IBD, RA, or psoriasis, we observed a substantial increase in PCVM among patients with the chronic infectious diseases studied, HIV and HCV. We suspect this is primarily due to change (decrease) in competing risk for non-cardiovascular mortality as treatment has changed for these conditions. HIV treatment strategies have changed markedly as a result of trials demonstrating the benefit of continuous and early post-diagnosis ART initiation, particularly SMART (2006) and START (2015)^19,20^. Since the publication of the SMART trial, increased use of ART has reduced the incidence of AIDS and deaths due to infectious causes, and prolonged the lifespan of people with HIV^1^. As people with HIV lived longer and underwent aging, their risk for mortality from cardiovascular disease also increased. A similar case can be made for HCV and the reduction in all-cause mortality and hepatocellular carcinoma incidence associated with direct-acting antivirals^2^. Notably, the individual-level risk of cardiac events remains elevated in these patients when compared to the general population, even with optimal infectious treatment^21,22^. As therapies in these conditions—and other chronic infectious states that may be associated with heightened cardiovascular risk—continue to evolve, monitoring changes in competing risks for non-infectious comorbidities such as CVDs will continue to be important^23^.

Notably, we observed substantial differences by race in PCVM in HIV and HCV. Observed PCVM among Black patients with either HIV or HCV was considerably higher than that among White patients with the same chronic infections. Numerous investigations have shown the contribution of social factors and bias on the part of healthcare providers to inferior outcomes experienced across the healthcare spectrum by Black Americans^24^. For example, previous work has demonstrated decreased statin uptake among communities of color when compared to White communities, possibly reflective of diminished access to subspecialty care^25^. Likewise, it is possible that barriers to effective HIV and/or HCV therapy for Black patients drive excess viral and immune activation, inflammation, and CVD risk^26^. These differences merit further study to curb what may be a substantial and accelerating excess in CVD burden and mortality among Black persons with HIV and/or HCV.

We also compare differences in subtypes of cardiovascular mortality in patients with CID in the first five and last five years of the period studied (1999–2003 and 2014–2018). These findings are most remarkable for a substantial decrease in death from ischemic heart disease (mirrored in the general population) and a moderate increase in the proportion of death from hypertensive diseases, the latter of which has been observed in recent years in the general population^27^. Increased attention given hypertensive disease in light of new data and changing guidelines that became available during the study period may have contributed to this trend in the general population. However, if this were the sole mechanism of increase, it is unclear why it would be greater in magnitude among those with CID, as observed in this investigation.

Although this analysis provided unprecedented scale for investigating CID-specific patterns in cardiovascular mortality over the past two decades, there are several limitations worth acknowledging. Many of these limitations relate to inherent limitations of death certificate data, which were used to determine primary and underlying/contributing causes of death. In order for a disease to be listed as a multiple cause of death, it must be recognized by the physician(s) attending the patient at the time of death. For several conditions studied, such as IBD and rheumatoid arthritis, a connection between the inflammatory disease of interest and the immediate cause of death (cardiovascular or otherwise) may not be readily apparent or the diagnosis known to providers filling out death certificates at the time of death. Given the scope of analyses and nature of the de-identified data set, we were unable to verify these diagnoses through individual chart review. However, previous work has demonstrated the validity of documented ICD codes (including the transition from ICD-9 to ICD-10) and diagnoses arrived at by expert reviewers blinded to the death certificate in large clinical cohorts, and the success at American death certificates of capturing a significant portion of those known to have chronic conditions such as SLE and RA^28–30^. Analysis of death certificate diagnosis and hospital discharge diagnosis in New York City conducted during this study period showed 85–90% concordance in 74,373 death certificates^31^. An additional limitation relates to our inability to investigate non-fatal outcomes (e.g., non-fatal cardiovascular events) as well as duration and intensity of exposures of interest. For instance, the systemic and vascular inflammatory damage from 30 years of poorly controlled RA is likely to differ considerably from the extent of cardiovascular damage and risk occurring in a person with recently diagnosed, mild RA. Indeed, our recent analysis of HF risk in different CIDs demonstrated increasing HF risk with increasing indicators of inflammatory burden across a number of CIDs^3^. However, even if this analysis has captured only a portion of those with chronic infectious and inflammatory conditions, we believe this still represents a substantial addition to present knowledge.

In conclusion, we observed substantial differences in patterns of proportionate cardiovascular mortality across a number of chronic infectious and inflammatory conditions at a population-level the United States from 1999 to 2018. Whereas the burden of cardiovascular mortality decreased in the general population and a number of chronic inflammatory conditions over this time period, it increased substantially for persons with HIV and HCV and remained stable/non-declining for persons with SLE. Further investigation of infectious and inflammatory condition-specific cardiovascular risks may serve clinical and translational purposes, informing cardiovascular risk stratification and therapy in these populations and informing mechanistic studies investigating immune-mediated cardiovascular disease pathogenesis.

## Methods

We used the publicly available detailed mortality database from the Centers for Disease Control and Prevention (CDC) Wide-Ranging Online Data for Epidemiologic Research (WONDER), described previously^4^. The database contains county-level national mortality and population data based on death certificates of all United States residents, which include underlying causes of death, up to 20 additional multiple causes of death (MCD), and demographic and geographic data. Causes of death are listed by international classification of diseases (ICD)-10 codes. Death certificates of all in-hospital and out-of-hospital deaths of U.S. residents are captured, while deaths of nonresidents are excluded.

The primary outcome for this study was the change in age-adjusted proportionate cardiovascular mortality (PCVM) from 1999 to 2018. Proportionate mortality was determined separately each year, for each chronic infectious or inflammatory condition, by dividing the age-adjusted death rate due to CVD by the age-adjusted death rate for all cause mortality in the same CID group. Age-adjusted all-cause death rates (denominator) are based on total deaths regardless of underlying cause but includes a specific CID as part of the multiple causes of death. Age-adjusted CVD death rates (numerator) are based on total deaths where CVD was the underlying cause and a CID was one of the multiple causes of death. Details on the calculation of age-adjusted death rates are available on the CDC WONDER webpage^32^.

Only adults aged 25 years and older at the time of death were included in this analysis. Those under age 25 were excluded as pediatric cardiovascular death tends to differ substantially from adult cardiovascular death in underlying cause. Age-adjustment was performed using the National Center for Health Statistics method with the 2000 U.S. standard population^33^. Age adjustment was necessary to allow comparisons across time and between disease types with differing underlying demographic characteristics. Cardiovascular mortality was defined as death with an underlying cause listed as ICD codes I00-I78 (which includes diagnoses such as ischemic heart diseases, cardiomyopathy, heart failure, and cerebral infarction, among others). Contributing causes of death, defined in CDC WONDER as “multiple causes of death”, were determined by ICD code as follows: B18.2, hepatitis C; B20–B24, HIV; K50–K51, inflammatory bowel disease; L40, psoriasis; M5–M6, rheumatoid arthritis; M32, systemic lupus erythematosus.

PCVM for each CID category at baseline and end of the study period was compared with that of the general population using ANOVA type III. Trends of PCVM for people with CID were compared to the general population using ordinary least squares regression with polynomial terms for time. To test if there were differences in rate of change in PCVM over time between CIDs, we checked if adding interaction terms between time and CID group improved model fit. We also tested for any break points in linear trajectories of PCVM per CID group using join point regression, with the ultimate goal of highlighting CID-specific periods of changing proportionate mortality (as well as potential CID-specific changes in disease natural and on-treatment history that may have informed these changes). For secondary analyses, we repeated the above analyses after stratifying by sex (male vs. female) and reported race (Black vs. White); additional stratification by non-binary gender variables or other race or ethnicity categories was not feasible due to incomplete or censored data for CID-specific comparisons.

We also assessed changes in the subtypes of CVD death within each CID group over time using graphical methods. Categories were hypertensive diseases (I10–I15), ischemic heart disease (I20–I25), pulmonary heart disease (I26–I28), cardiomyopathy and heart failure (I42 and I50), cerebrovascular disease (I60–I69), and other cardiovascular death (all I00–I99 diagnoses other than I10–I15, I20–I28, I42, I50, and I60–69). We then compared average age-adjusted death rates due to a CVD subtype among individuals with any of the six CIDs in the first five years (1999–2003) to the average in the last 5 years (2014–2018). We chose to compare the first 5 and last 5 years combined as we were unable to calculate stable yearly estimates of CVD subtype-specific mortality for each CID due to limited power.

All analyses were conducted in R 4.0.3 with figures generated using the ggplot2 package^34,35^. Joinpoint regression was done using the segmented package^36^. Model fitting to identify the parsimonious model was guided by likelihood ratio tests and Akaike Information Criteria, with lower numbers indicating better model parsimony.

### Human research statement

All methods were carried out in accordance with relevant local, state, national, and institutional guidelines and regulations for human research.

## Supplementary Information

Supplementary Information.

## Data Availability

All data cited in this text is obtained from the Centers for Disease Control and Prevention’s WONDER Multiple Cause of Death dataset, freely available online at https://wonder.cdc.gov/mcd.html.
