# Supplementary material for: Changes in proportionate cardiovascular mortality in patients with chronic infectious and inflammatory conditions in the United States, 1999–2018
Source: Sci Rep. 2021 Dec 14;11:23985. doi: 10.1038/s41598-021-03407-4 (PMC8671419; doi:10.1038/s41598-021-03407-4)
Supplement: Supplementary file 1 — Supplementary Information. [file 41598_2021_3407_MOESM1_ESM.docx]

**eFigure 1:** Proportionate mortality over time by disease and sex, 1999-2018


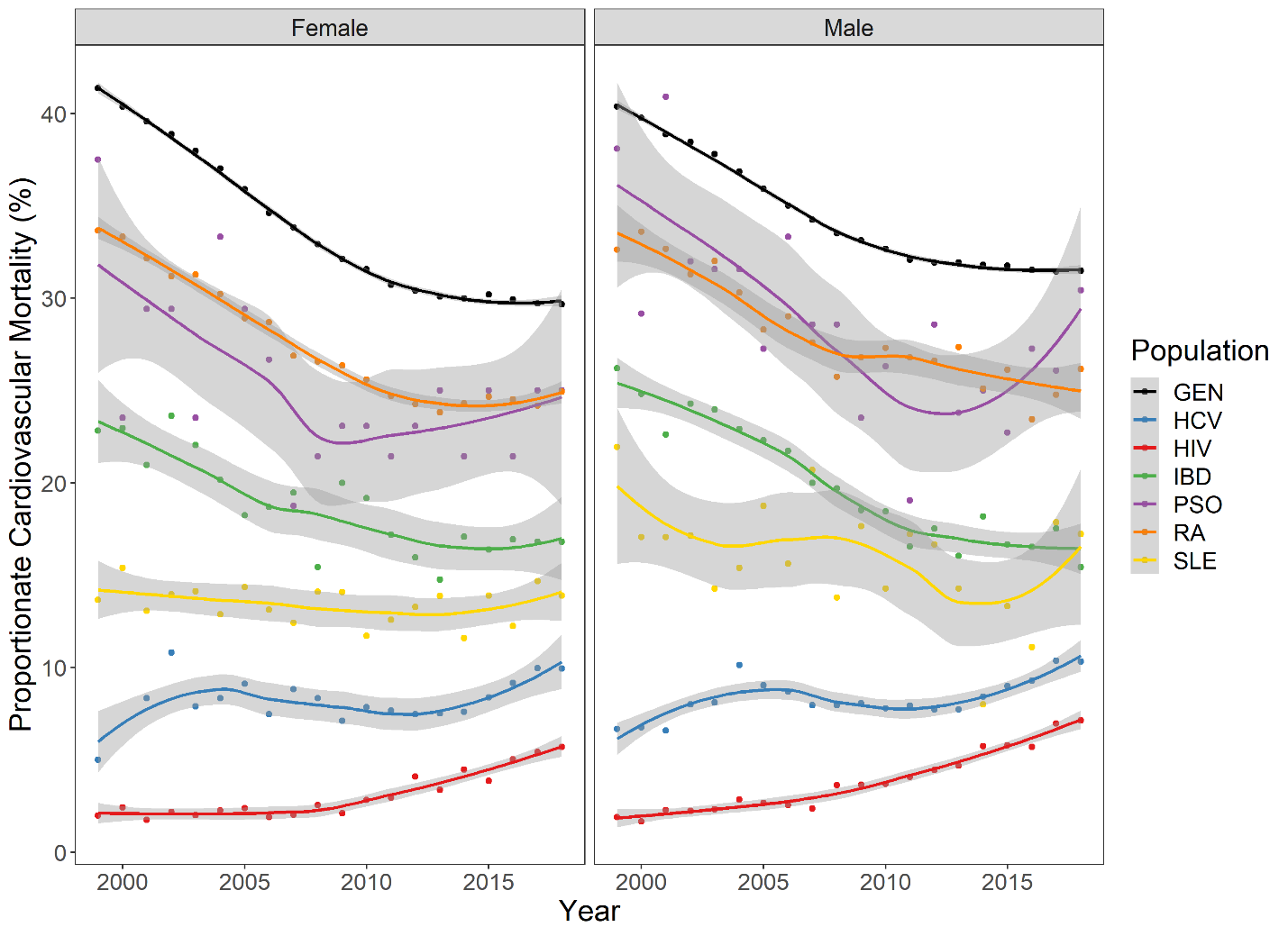


**eFigure 1:**

GEN: General population. HCV: Chronic hepatitis C virus, ICD-10 code B18.2. HIV: Human immunodeficiency virus, ICD-10 codes B20-24. IBD: Inflammatory bowel disease, ICD-10 codes K50-51. PSO: Psoriasis, ICD-10 code L40. RA: Rheumatoid arthritis, ICD-10 codes M5-M6. SLE: Systemic lupus erythematous, ICD-10 code M32.

eFigure 2: Proportionate mortality over time by disease and reported race, 1999-2018


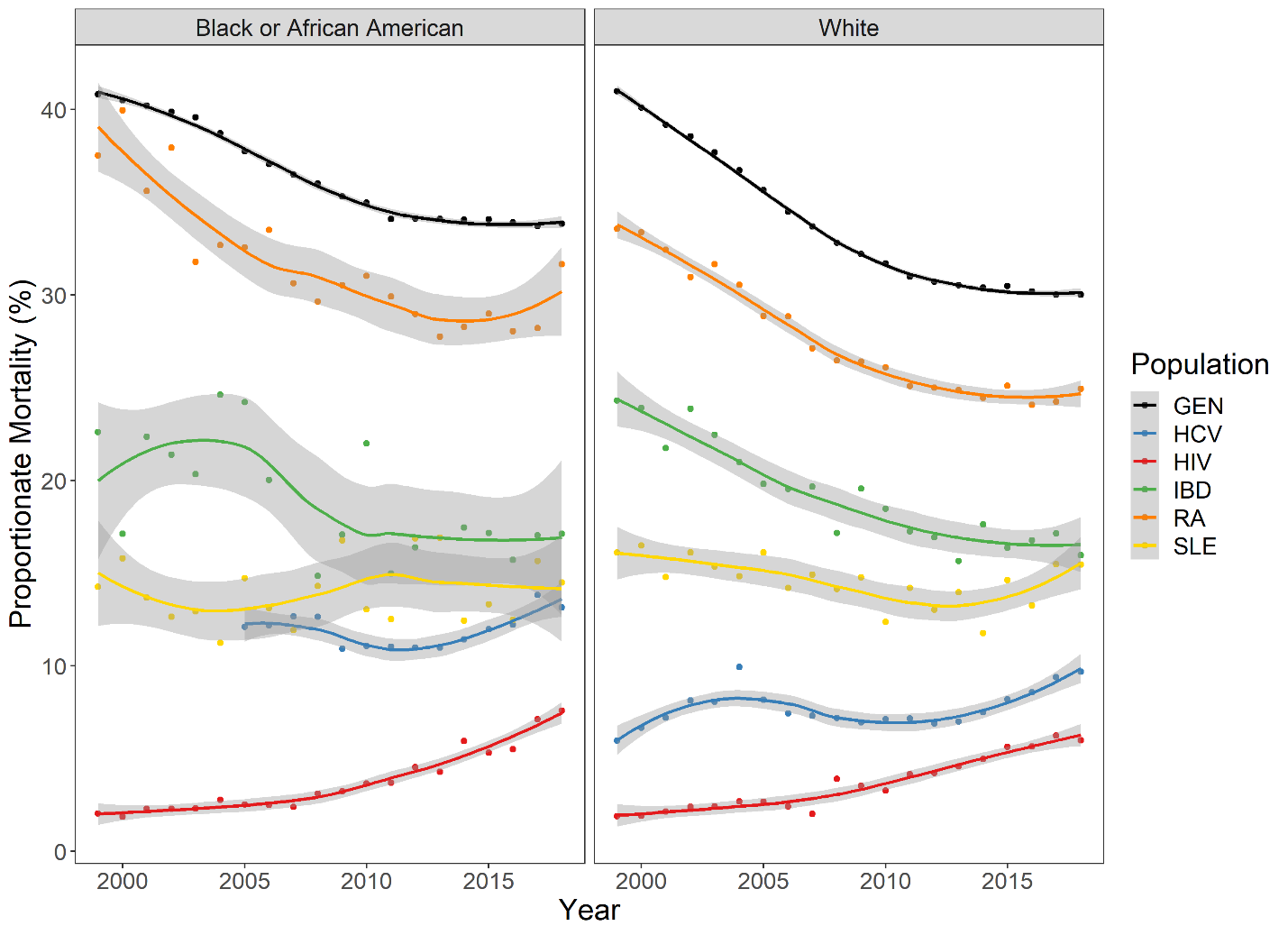


**eFigure 2:**

GEN: General population. HCV: Chronic hepatitis C virus, ICD-10 code B18.2. HIV: Human immunodeficiency virus, ICD-10 codes B20-24. IBD: Inflammatory bowel disease, ICD-10 codes K50-51. PSO: Psoriasis, ICD-10 code L40. RA: Rheumatoid arthritis, ICD-10 codes M5-M6. SLE: Systemic lupus erythematous, ICD-10 code M32.

**eTable 1:** Difference in PCVM by Race and CID Group

| Group | PCVM in 1999^a^ | | | PCVM in 2018 | | |
| --- | --- | --- | --- | --- | --- | --- |
|  | Black | White | Difference^b^ | Black | White | Difference^b^ |
| HIV | 2.0 | 1.9 | -0.7 (-1.6 to 0.1) | 7.6 | 6.0 | 1.0 (0.1 to 1.8) |
| HCV^a^ | 12.1 | 8.2 | 3.6 (2.7 to 4.5) | 13.2 | 9.7 | 4.7 (3.8 to 5.6) |
| IBD | 22.6 | 24.3 | -1.2 (-2.1 to -0.3) | 17.1 | 16 | 0.5 (-0.4 to 1.4) |
| RA | 37.5 | 33.6 | 3.2 (2.3 to 4.1) | 31.7 | 24.9 | 4.9 (4.0 to 5.8) |
| SLE | 14.3 | 16.1 | -1.5 (-2.4 to -0.6) | 14.5 | 15.5 | 0.2 (-0.7 to 1.1) |

**eTable 1:**

Notes: ^a^ - for HCV, PCVM is from 2005 and not 1999 since data for Black individuals before 2005 were censored for too few cases. ^b^ - Differences between those self-reporting as Black and White were estimated using a linear model with polynomial terms for time (up to time^3^) and interaction terms between group and time (time and time^2^), between race and time, and between group and race.
